# Development, Validation, and Evaluation of a Simple Machine Learning Model to Predict Cirrhosis Mortality

**DOI:** 10.1001/jamanetworkopen.2020.23780

**Published:** 2020-11-03

**Authors:** Fasiha Kanwal, Thomas J. Taylor, Jennifer R. Kramer, Yumei Cao, Donna Smith, Allen L. Gifford, Hashem B. El-Serag, Aanand D. Naik, Steven M. Asch

**Affiliations:** 1 Section of Gastroenterology and Hepatology, Department of Medicine, Baylor College of Medicine, Houston, Texas; 2 Health Services Research, Department of Medicine, Baylor College of Medicine, Houston, Texas; 3 Veterans Affairs (VA) Health Services Research and Development Service Center for Innovations in Quality, Effectiveness, and Safety, Houston, Texas; 4 Michael E. DeBakey VA Medical Center, Houston, Texas; 5 Center for Innovation to Implementation, VA Palo Alto Health Care System, Palo Alto, California; 6 Department of Medicine, VA Boston Healthcare System, Boston University, Boston, Massachusetts; 7 Department of Health Law, Policy, and Management, VA Boston Healthcare System, Boston University, Boston, Massachusetts; 8 Section of Geriatrics and Palliative Medicine, Department of Medicine, Baylor College of Medicine, Houston, Texas; 9 Division of Primary Care and Population Health, Department of Medicine, Stanford University, Stanford, California

## Abstract

**Importance:**

Machine-learning algorithms offer better predictive accuracy than traditional prognostic models but are too complex and opaque for clinical use.

**Objective:**

To compare different machine learning methods in predicting overall mortality in cirrhosis and to use machine learning to select easily scored clinical variables for a novel cirrhosis prognostic model.

**Design, Setting, and Participants:**

This prognostic study used a retrospective cohort of adult patients with cirrhosis or its complications seen in 130 hospitals and affiliated ambulatory clinics in the integrated, national Veterans Affairs health care system from October 1, 2011, to September 30, 2015. Patients were followed up through December 31, 2018. Data were analyzed from October 1, 2017, to May 31, 2020.

**Exposures:**

Potential predictors included demographic characteristics; liver disease etiology, severity, and complications; use of health care resources; comorbid conditions; and comprehensive laboratory and medication data. Patients were randomly selected for model development (66.7%) and validation (33.3%). Three different statistical and machine learning methods were evaluated: gradient descent boosting, logistic regression with least absolute shrinkage and selection operator (LASSO) regularization, and logistic regression with LASSO constrained to select no more than 10 predictors (partial pathway model). Predictor inclusion and model performance were evaluated in a 5-fold cross-validation. Last, the predictors identified in the most parsimonious (the partial path) model were refit using maximum-likelihood estimation (Cirrhosis Mortality Model [CiMM]), and its predictive performance was compared with that of the widely used Model for End Stage Liver Disease with sodium (MELD-Na) score.

**Main Outcomes and Measures:**

All-cause mortality.

**Results:**

Of the 107 939 patients with cirrhosis (mean [SD] age, 62.7 [9.6] years; 96.6% male; 66.3% white, 18.4% African American), the annual mortality rate ranged from 8.8% to 15.3%. In total, 32.7% of patients died within 3 years, and 46.2% died within 5 years after the index date. Models predicting 1-year mortality had good discrimination for the gradient descent boosting (area under the receiver operating characteristics curve [AUC], 0.81; 95% CI, 0.80-0.82), logistic regression with LASSO regularization (AUC, 0.78; 95% CI, 0.77-0.79), and the partial path logistic model (AUC, 0.78; 95% CI, 0.76-0.78). All models showed good calibration. The final CiMM model with machine learning–derived clinical variables offered significantly better discrimination than the MELD-Na score, with AUCs of 0.78 (95% CI, 0.77-0.79) vs 0.67 (95% CI, 0.66-0.68) for 1-year mortality, respectively (DeLong *z = *17.00; *P* < .001).

**Conclusions and Relevance:**

In this study, simple machine learning techniques performed as well as the more advanced ensemble gradient boosting. Using the clinical variables identified from simple machine learning in a cirrhosis mortality model produced a new score more transparent than machine learning and more predictive than the MELD-Na score.

## Key Points

**Question:**

Can a blended approach that uses clinical variables selected from machine learning to develop traditional prognostic models improve the accuracy of prediction while addressing challenges related to interpretability?

**Findings:**

In a prognostic study including a cohort of 107 939 patients with cirrhosis, simple machine learning techniques performed as well as the more advanced ensemble gradient boosting techniques. Using the clinical variables identified from simple machine learning in a cirrhosis mortality model produced a new score more predictive than the traditional Model for End Stage Liver Disease with sodium.

**Meaning:**

These findings suggest that this blended approach can improve data-driven risk prognostication through the development of new scores that are both more transparent and actionable than machine learning and more predictive than traditional risk scores.

## Teaser

This cohort study compares different machine learning methods in predicting overall mortality in cirrhosis and uses machine learning to select easily scored clinical variables for a novel prognostic model in patients with cirrhosis.

## Introduction

Risk stratification is at the core of medical practice. Risk prediction scores now routinely guide treatment across a range of medical decisions from anticoagulation^1,2^ to lowering of cholesterol levels^3^ to life-sustaining intensive care.^4^ The most widely used scores include a limited number of easily measured variables, allowing for transparent calculation and interpretability but constraining their prognostic performance.

Machine learning techniques have the potential to improve prognostication. These techniques incorporate a large array of predictors in a nonlinear pattern and use multiple interactions to enhance accuracy. However, their many variables and the complexity of scoring rules hinder their implementation in all but the most advanced informatics settings. Even when informatics infrastructure supports them, the “black box” nature of the algorithms means they are inherently unexplainable to clinicians and patients.^5^ A blended strategy that builds on the strengths of machine learning to develop simpler, clinically explainable risk scores may optimize the trade-off between accuracy vs interpretability and also facilitate subsequent implementation.

Cirrhosis is a high-risk common condition with a progressive clinical course. The most widely used cirrhosis prognostic models such as the Model for End Stage Liver Disease with sodium (MELD-Na) or Child-Turcotte-Pugh,^6,7^ are disease-specific scores but may have modest discriminative ability for overall mortality. Other prognostic scores in cirrhosis are mostly applicable in restricted settings, such as scores predicting short-term risk of dying during or after hospitalization.^8,9^ None of these scores account for a wide range of clinical and psychosocial factors that are likely to be associated with mortality in cirrhosis. Machine learning techniques have been used to help fill these gaps for cirrhosis but have not seen widespread use.^10^

We performed a prognostic study using data from a retrospective cohort of patients with cirrhosis seen at 130 US Department of Veterans Affairs (VA) hospitals in the United States. We first developed and compared 3 machine learning algorithms with varying levels of complexity and range of variables that predicted risk of mortality in cirrhosis. To achieve a balance among accuracy, interpretability, and feasibility, we then developed and validated a blended model (Cirrhosis Mortality Model [CiMM]) that used the variables selected from machine learning algorithms and implemented them in an accessible platform. Last, we compared the CiMM with the MELD-Na score to determine whether it meaningfully improved mortality risk predictions in cirrhosis over this widely used model.

## Methods

### Data Sources

We used data from the national VA Corporate Data Warehouse that includes all laboratory test results, pharmacy, inpatient and outpatient procedures, and diagnosis codes for patients using the VA for health care. We also used the VA Purchased Care database of services paid by but rendered outside the VA. We obtained date of death from the VA Vital Status file. This study was approved by the institutional review board of Baylor College of Medicine, Houston, Texas, which waived the need for informed consent, and followed the Transparent Reporting of a Multivariable Prediction Model for Individual Prognosis or Diagnosis (TRIPOD) reporting guideline.

### Study Cohort

Our cohort included patients with cirrhosis who were seen in ambulatory clinics at 130 VA hospitals from October 1, 2011, to September 30, 2015. We included patients if they had at least 2 instances of cirrhosis or cirrhosis complications codes or at least 1 code for cirrhosis or complications with at least 1 filled prescription of spironolactone (≥100 mg for ascites), rifaximin, or lactulose (for encephalopathy) after a cirrhosis diagnosis. These case ascertainment strategies were found to have high positive predictive value (86%-93%) for the presence of cirrhosis in the patients’ medical records.^7,11,12^

We selected the first clinic visit at or after meeting cohort entry criteria as the index date for follow-up (eMethods 1 in the Supplement). We excluded patients younger than 18 or older than 90 years or who received a liver transplant before the index date. We acquired data through December 31, 2018, to ascertain end points.

### Variable Selection

For the dependent variable, we obtained all-cause mortality data from VA Vital Status File that combines information from the VA Death File, VA Compensation and Pension Benefits, Medicare, and Social Security and has a sensitivity of 98.3% and specificity of 99.8% relative to the National Death Index.^13^ We based the selection of predictor variables on a priori hypotheses guided by literature and clinical knowledge as well as easy availability in the electronic health record. Sociodemographic variables included age, sex, race/ethnicity, rural status, marital status, means test (financial assessment), and enrollment priority level.^14^ eTable 1 in the Supplement gives the *International Classification of Diseases, Ninth Revision, Clinical Modification* (*ICD-9-CM*), *Current Procedural Terminology*, and drug class codes used to define predictor variables.

We defined hepatitis C virus (HCV) based on any evidence of positive HCV ribonucleic acid test result,^15^ hepatitis B virus (HBV) based on any positive finding for hepatitis B surface antigen,^16^ and alcohol-related liver disease based on at least 1 *ICD-9-CM* code for alcohol use disorders at any time or positive 3-Item Alcohol Use Disorders Identification Test scores (≥4 in men and ≥3 in women) within 1 year before the index date. For HCV, we also determined whether patients had achieved sustained virologic response.^17^ We identified nonalcoholic steatohepatitis as the possible etiology of cirrhosis for patients without any other cause who had type 2 diabetes or body mass index (calculated as weight in kilograms divided by height in meters squared) of greater than 30 before diagnosis of cirrhosis.^18^

We extracted data for serum levels of bilirubin, sodium, and creatinine and international normalized ratio performed within 1 year before and closest to the index date. We also combined them to derive MELD-Na scores.^12^ Other liver disease factors included serum levels of albumin, hemoglobin, alanine aminotransferase (ALT), and aspartate aminotransferase (AST), AST:ALT ratio, and platelet counts within 1 year before the index date. We defined type and number of cirrhosis complications and infections.^19,20^

At least 40% of patients with cirrhosis may have comorbidities that increase mortality.^21,22^ We defined medical conditions using the cirrhosis-specific comorbidity (CirCom) score,^21^ which includes chronic obstructive pulmonary disease, history of myocardial infarction, peripheral artery disease, epilepsy, drug abuse, heart failure, nonmetastatic or hematological cancer, metastatic cancer, and chronic kidney disease. Other health conditions included diabetes, history of infection, depression, anxiety, and alcohol use. Table 1 and eTable 1 in the Supplement describe the codes used for CirCom scores and other conditions.

**Table 1.** Baseline Characteristics of 107 939 Patients With Cirrhosis

Characteristic	Data^a^
Age, mean (SD), y	62.7 (9.6)
Race/ethnicity	
White	71 563 (66.3)
Black	19 852 (18.4)
Hispanic	6376 (5.9)
Other	3005 (2.8)
Sex	
Female	3623 (3.4)
Male	104 316 (96.6)
Marital status	
Divorced or separated	47 981 (44.5)
Married	45 792 (42.4)
Single or never married	14 020 (13.0)
Etiology of cirrhosis	
HCV infection alone	14 286 (13.2)
HCV and alcohol	26 011 (24.1)
Alcohol alone	34 112 (31.6)
Nonalcoholic steatohepatitis	29 140 (27.0)
HBV infection	3427 (3.2)
MELD-Na score^b^	
<10	36 600 (33.9)
10-20	29 442 (27.3)
>20	6329 (5.9)
Missing	35 568 (32.9)
Cirrhosis complications	
Hepatic encephalopathy	21 556 (20.0)
Ascites	21 770 (20.2)
Varices	17 631 (16.3)
Hepatocellular cancer	8150 (7.6)
Laboratory test results, mean (SD)	
Sodium level, mEq/L	137.7 (3.8)
Creatinine level, mg/dL	1.2 (1.0)
Bilirubin level, mg/dL	1.6 (2.7)
Albumin level, g/dL	3.5 (0.7)
Platelet count, ×10^3^/μL	166.5 (92.7)
Hemoglobin level, g/dL	13.0 (2.3)
Physical health conditions	
Diabetes	54 137 (50.2)
Chronic obstructive pulmonary disease	17 326 (16.1)
Heart failure	11 332 (10.5)
Cancer	18 164 (16.8)
Chronic kidney disease	10 872 (10.1)
CirCom score^c^	
0	25 649 (23.8)
1 + 0	28 853 (26.7)
1 + 1	20 362 (18.9)
3 + 0	5813 (5.4)
3 + 1	23 807 (22.1)
5 + 0	109 (0.1)
5 + 1	3346 (3.1)
Use of health care resources	
Hospitalization due to any cause in past year	44 143 (40.9)
Hospitalization with primary diagnosis of cirrhosis in past year	10 560 (9.8)
≥1 Emergency department visit in the past year	46 450 (43.0)
≥3 Outpatient visits in the past year	98 173 (91.0)

Abbreviations: CirCom, cirrhosis-specific comorbidity score; HBV, hepatitis B virus; HCV, hepatitis C virus.

SI conversion factors: To convert albumin to g/L, multiply by 10.0; bilirubin to μmol/L, multiply by 17.104; creatinine to μmol/L, multiply by 88.4; hemoglobin to g/L, multiply by 10.0; platelet count to ×10^9^/L, multiply by 1.0; sodium to mmol/L, multiply by 1.0.

^a^ Unless otherwise indicated, data are expressed as number (percentage) of patients. Owing to missing data, percentages may not total 100.

^b^ Higher scores indicate more severe liver disease.

^c^ The CirCom score uses a specific set of *International Statistical Classification of Diseases and Related Health Problems, Tenth Revision*, codes to define the conditions. We mapped these to *International Classification of Diseases, Ninth Revision, Clinical Modification*, codes to define clinical conditions (eTable 1 in the Supplement). We used the Academy of Healthcare Research and Quality Clinical Classifications Software to define the conditions that were not part of the CirCom score (eg, diabetes, depression, anxiety, and alcohol use). Nonmetastatic cancer, metastatic cancer, hematologic cancer, substance abuse other than alcoholism, epilepsy, acute myocardial infarction, heart failure, peripheral arterial disease, chronic obstructive pulmonary disease, and chronic kidney disease were pulled using most recent inpatient or outpatient diagnoses given in the 5 years before index date. The CirCom score was calculated by the algorithm developed and validated by Jepsen et al.^21^

We used outpatient prescription files to identify medication classes selected based on frequency of use among our cohort or known associations with cirrhosis outcomes. We also extracted information on other therapies for cirrhosis complications, such as endoscopic variceal ligation, paracentesis, and transjugular intrahepatic portosystemic shunts in the year before the index date.

We included the most current values of pulse, blood pressure, respiratory rate, and body mass index recorded within 1 year before and closest to the index date.^14^ We also included data on history of smoking and the most current levels of total, low-density lipoprotein, and high-density lipoprotein cholesterol because they are associated with all-cause mortality in the general population. Last, we included history of hospitalization (both liver and all-cause hospitalization), number of outpatient visits, and whether the patient sought emergency care in the year before or any time before the index visit.

### Statistical Analysis

Data were analyzed from October 1, 2017, to May 31, 2020. Few laboratory values were missing in more than 5% of patients. We imputed data using a machine learning–based imputation, MissForest (eMethods 2 in the Supplement).^23,24^ We structured the data to evaluate risk of mortality at yearly time horizons using discrete time-to-event methods.^25,26,27^ Discrete time-to-event methods avoid issues with the proportional hazards assumption^28,29^ and use person-periods to allow for nonproportional changes in risk of mortality across time within each patient.^27^ We used this approach because several candidate predictors violated the proportional hazards assumption. Patients could have 1 to 8 years of follow-up before death or censoring.

#### Prediction Models

We developed and compared 3 models. First, we used extreme gradient descent boosting,^30,31^ which accounts for higher-order, nonlinear interactions in a variety of data types, including binary and continuous variables, and selects variables while training. Second, we used logistic regression with least absolute shrinkage and selection operator (LASSO) regularization, which is a technique that alters the model fitting to select only a subset of predictors based on gradient descent instead of relying on *P* values (eMethods 3 in the Supplement).^32^ The first 2 methods used the full set of predictors (ie, full models). In the third step, we constrained the logistic regression model to the top features set, including a maximum of 10 individual factors (partial path model),^5^ because most people can interpret, at most, 7 to 10 entities at a time.^33^

#### Derivation and Validation

We split the cohort into a derivation (66.7% of the data) and a holdout validation set (33.3% of the data); the same holdout set was used across all models fit. The derivation set was divided randomly into 5 equal subsets, preserving the same event rate in each subset. We combined 4 of the subsets for derivation and reserved the remaining subset as internal validation.

Subsequent to training the models, we estimated discrimination via the area under the receiver operating characteristics curve (AUC) in the validation set. We computed AUCs specific to each time horizon (discrimination).^34,35^ We evaluated calibration^36,37^ with Brier scores and with visual assessments of calibration curves for each time horizon. Brier scores can be interpreted as how far the prediction is from the observed estimate^37^; lower scores indicate better calibration.

To allow easy applicability, we refitted the predictors included in the best-performing model using maximum-likelihood discrete time-to-event logistic regression estimation (CiMM). We converted the odds ratios to relative risk (RR) ratios (2-sided *P* < .05 indicated statistical significance).^38^ In the last step, we compared the performance of the CiMM with that of the MELD-Na score.

### Risk Scoring and Risk Stratification

To show how the CiMM may be applied for risk stratification, we selected a range of clinically plausible values on all predictors and applied the CiMM to these plausible patient profiles. We retained the predicted risk on a probability scale between no risk (0.00) and near complete risk (>0.99).

## Results

### Demographic Characteristics

We identified 107 939 patients with cirrhosis (Table 1). The mean (SD) age of patients was 62.7 (9.6) years (96.6% male and 3.4% female); 66.3% were White; 18.4% were Black; and 42.4% were married. Most patients (68.9%) had HCV- or alcohol-related cirrhosis; 26.9% had nonalcoholic steatohepatitis cirrhosis. Approximately one-third (33.1%) had a MELD-Na score of 10 or higher; 20.2% had ascites, and 20.0% had hepatic encephalopathy. Participants had high rates of history of drug or alcohol abuse (41.4%), chronic obstructive pulmonary disease (16.1%), and heart failure (10.5%) in the past year. In total, 40.9% of the cohort was hospitalized for any cause in the year before the index date. eTable 2 in the Supplement shows the complete set of variables used in the full models.

**Figure 1.**
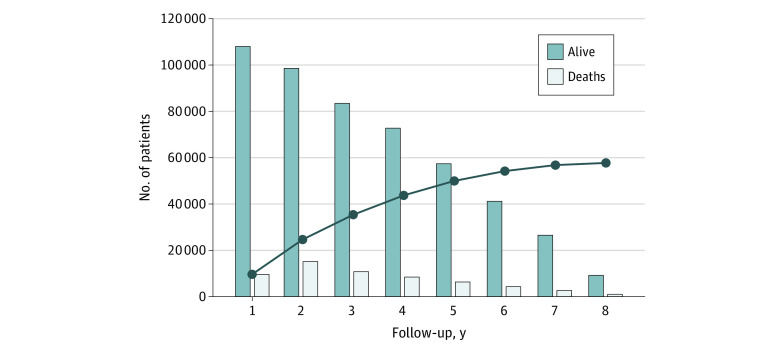
Annual and Cumulative Incidence of All-Cause Mortality Includes 107 939 participants at inception. Annual all-cause mortality was 8.8% at 1 year, 15.3% at 2 years, 12.8% at 3 years, 11.5% at 4 years, 10.9% at 5 years, 10.4% at 6 years, 9.8% at 7 years, and 10.3% at 8 years. Cumulative all-cause mortality was 8.8% at 1 year, 22.8% at 2 years, 32.7% at 3 years, 40.4% at 4 years, 46.2% at 5 years, 50.2% at 6 years, 52.6% at 7 years, and 53.4% at 8 years.

Figure 1 displays the annual and cumulative incidence of all-cause mortality. The annual mortality rate ranged from 8.8% to 15.3%. In total, 32.7% of patients died within 3 years, and 46.2% died within 5 years after the index date.

### Prediction Model Performance

Model discrimination was high across the 3 approaches (Table 2). For the full gradient boosting model, the AUC for predicting 1-year mortality was 0.81 (95% CI, 0.80-0.82). For the full discrete time-to-event logistic model with LASSO, the AUC for 1-year mortality was slightly lower at 0.78 (95% CI, 0.77-0.79). Constraining the logistic regression model to include the subset of important variables (partial path logistic) resulted in AUCs that were similar (0.78; 95% CI, 0.76-0.78) to the full logistic model with LASSO. Overall, mortality predictions for year 2 onward showed similar trends, although the overall discrimination fell as time elapsed (years 2-8 AUCs: full gradient boosting range, 0.78-0.72; full discrete logistic regression range, 0.76-0.69; partial discrete logistic regression range, 0.76-0.67).

**Table 2.** Discrimination and Calibration of 3 Modeling Approaches

Method	No. of patients	Person-year	AUC (95% CI) discrimination	Brier score calibration (95% CI)
Extreme gradient boosting	32 437	1	0.81 (0.80-0.82)	0.07 (0.07-0.07)
	29 603	2	0.78 (0.77-0.79)	0.11 (0.11-0.11)
	25 084	3	0.74 (0.73-0.75)	0.10 (0.10-0.11)
	21 908	4	0.72 (0.71-0.73)	0.10 (0.09-0.10)
	17 309	5	0.72 (0.71-0.73)	0.09 (0.09-0.10)
	12 341	6	0.72 (0.70-0.73)	0.09 (0.08-0.09)
	7940	7	0.71 (0.69-0.73)	0.09 (0.08-0.09)
	2715	8	0.72 (0.69-0.75)	0.09 (0.07-0.10)
Full discrete time-to-event logistic regression with LASSO	32 437	1	0.78 (0.77-0.79)	0.07 (0.07-0.08)
	29 603	2	0.76 (0.75-0.77)	0.11 (0.11-0.12)
	25 084	3	0.72 (0.71-0.73)	0.10 (0.10-0.11)
	21 908	4	0.70 (0.69-0.71)	0.10 (0.09-0.10)
	17 309	5	0.69 (0.68-0.71)	0.09 (0.09-0.10)
	12 341	6	0.69 (0.67-0.70)	0.09 (0.08-0.09)
	7940	7	0.69 (0.68-0.71)	0.09 (0.08-0.09)
	2715	8	0.69 (0.66-0.72)	0.09 (0.08-0.10)
Partial discrete time-to-event logistic regression with LASSO	32 437	1	0.78 (0.76-0.78)	0.07 (0.07-0.08)
	29 603	2	0.76 (0.74-0.76)	0.12 (0.11-0.12)
	25 084	3	0.71 (0.70-0.72)	0.10 (0.10-0.11)
	21 908	4	0.68 (0.67-0.69)	0.10 (0.09-0.10)
	17 309	5	0.67 (0.66-0.69)	0.09 (0.09-0.10)
	12 341	6	0.67 (0.65-0.68)	0.09 (0.08-0.09)
	7940	7	0.68 (0.66-0.70)	0.09 (0.08-0.09)
	2715	8	0.67 (0.64-0.70)	0.09 (0.08-0.10)

Abbreviations: AUC, area under the receiver operating characteristics curve; LASSO, least absolute shrinkage and selection operator.

The Brier scores ranged from 0.07 (95% CI, 0.07-0.07) to 0.11 (95% CI, 0.11-0.11) using gradient boosting, with the full and partial path discrete time-to-event logistic regression models all indicating good calibration (Table 2). eFigures 1 and 2 in the Supplement show the discrimination and calibration slopes for the gradient boosting, full logistic, and partial path models.

### Prediction Model Interpretation

Given similar performance characteristics across different models and based on a priori considerations, we retained the partial path discrete time-to-event logistic regression model because it can be interpreted and feasibly implemented in different clinical settings. We modeled the selected predictors using maximum-likelihood discrete time-to-event logistic regression to develop the CiMM.

**Figure 2.**
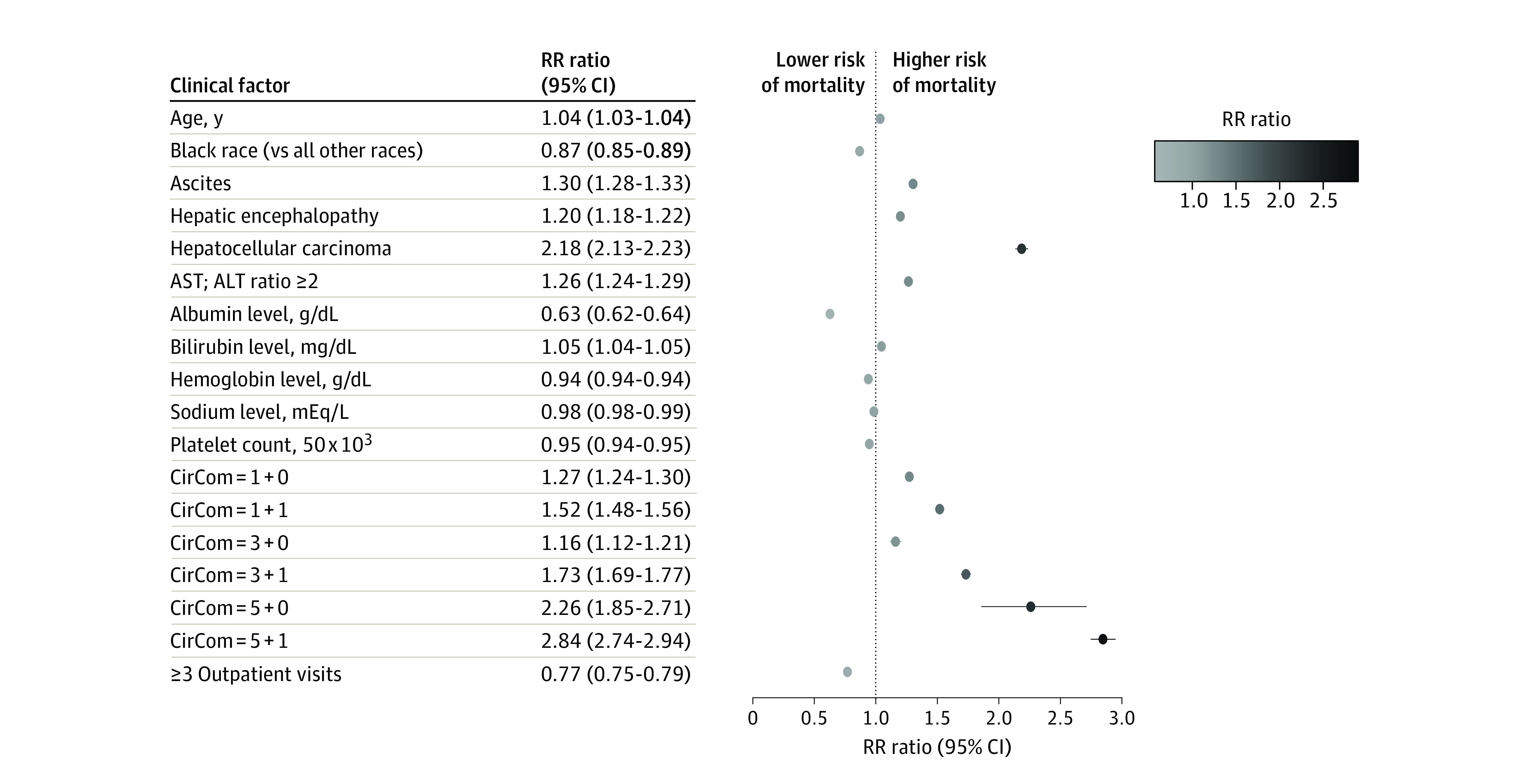
Associations Between Clinical Factors Included in Cirrhosis Mortality Model and Time to Death ALT indicates alanine aminotransferase; AST, aspartate aminotransferase; CirCom, cirrhosis-specific comorbidity score; and RR, relative risk.

Figure 2 shows the visual range of coefficients in the CiMM. Older age (RR ratio per 1-year increase, 1.04; 95% CI, 1.03-1.04), higher bilirubin level (RR ratio per additional unit, 1.05; 95% CI, 1.04-1.05), an AST:ALT ratio greater than 2 (RR ratio, 1.26; 95% CI, 1.24-1.29), hepatic encephalopathy (RR ratio, 1.20; 95% CI, 1.18-1.22), ascites (RR ratio, 1.30; 95% CI, 1.28-1.33), and hepatocellular carcinoma (RR ratio, 2.18; 95% CI, 2.13-2.23) were significantly associated with higher risk of mortality. Compared with patients with a CirCom score of 0 (no comorbidity), the RR ratio ranged from 1.27 (95% CI, 1.24-1.30) for patients with CirCom score of 1 + 0 (≥1 of the following: chronic obstructive pulmonary disease, peripheral artery disease, epilepsy, drug abuse, or heart failure) to 2.84 (95% CI, 2.74-2.94) for patients with CirCom score of 5 + 1 (active metastatic cancer and 1 more comorbidity).

Black participants had lower risk (RR ratio, 0.87; 95% CI, 0.85-0.89) than patients in other racial groups. Greater connection to health care (RR ratio, 0.77; 95% CI, 0.75-0.79) was associated with lower mortality. Higher albumin level (RR ratio per additional unit, 0.63; 95% CI, 0.62-0.64), higher hemoglobin level (RR ratio per additional unit, 0.94; 95% CI, 0.94-0.94), higher sodium level (RR ratio per additional unit, 0.98; 95% CI, 0.98-0.99), and higher platelet count (RR ratio per additional 50 units, 0.95; 95% CI, 0.94-0.95) decreased risk for mortality.

### Comparative Analysis of CiMM and MELD-Na

Table 3 compares the CiMM’s performance with that of the MELD-Na score. The AUCs with the CiMM were 0.78 (95% CI, 0.77-0.79) for 1-year mortality, 0.76 (95% CI, 0.75-0.77) for 2-year mortality, and 0.72 (95% CI, 0.71-0.73) for 3-year mortality. The corresponding AUCs for MELD-Na were 0.67 (95% CI, 0.66-0.68) for 1-year mortality, 0.65 (95% CI, 0.64-0.66) for 2-year mortality, and 0.61 (95% CI, 0.60-0.62) for 3-year mortality; *P* < .001 for each discrete year and remained so after false discovery rate adjustment (DeLong *z = *17.00). The Brier scores for the MELD-Na predictions ranged from 0.08 (95% CI, 0.07-0.08) to 0.13 (95% CI, 0.12-0.13). Across all ranges of false-positive rates (AUC), the mean sensitivity of the CiMM was 10% to 11% higher than that for the MELD-Na score for all years.

**Table 3.** Comparison of Discrimination Between the CiMM and MELD-Na Score^a^

Person-year	No. of patients	AUC (95% CI)		DeLong test (*z* statistic)	*P* value
		CiMM	MELD-Na score		
1	107 939	0.78 (0.77-0.79)	0.67 (0.66-0.68)	17.00	<.001
2	98 419	0.76 (0.75-0.77	0.65 (0.64-0.66)	21.29	<.001
3	83 368	0.72 (0.71-0.73)	0.61 (0.60-0.62)	17.44	<.001
4	72 665	0.69 (0.68-0.70)	0.60 (0.59-0.61)	14.54	<.001
5	57 301	0.69 (0.67-0.70)	0.59 (0.58-061)	12.79	<.001
6	41 087	0.68 (0.66-0.69)	0.58 (0.56-0.60)	10.95	<.001
7	26 441	0.69 (0.67-0.71)	0.60 (0.58-0.62)	7.29	<.001
8	9085	0.68 (0.65-0.71)	0.57 (0.53-0.61)	4.47	<.001

Abbreviations: AUC, area under the receiver operating characteristics curve; CiMM, cirrhosis mortality model; MELD-Na, Model for End Stage Liver Disease with sodium.

^a^ Maximum-likelihood model used the predictors identified from partial discrete time-to-event logistic regression model with least absolute shrinkage and selection operator.

### Risk Scoring and Risk Stratification

eTable 3 in the Supplement shows the predicted risk of death across different patient profiles. For example, a 55-year-old patient with a serum bilirubin level of 2 mg/dL (to convert to μmol/L, multiply by 17.104), platelet count of 150 × 10^3^/μL, albumin and hemoglobin values within the reference range, ascites but no other cirrhosis complications, and a history of myocardial infarction (CirCom 1) has a 12% risk of death within 1 year. In contrast, a 65-year-old patient with a bilirubin level of 4 mg/dL, platelet count 150 × 10^3^/μL, albumin level of 2.5 g/dL (to convert to g/L, multiply by 10.0), hemoglobin level of 10.5 g/dL (to convert to g/L, multiply by 10.0), ascites, chronic kidney disease, and a hematological cancer (CirCom 3 + 1) has a 66% risk of death within 1 year. eTable 4 in the Supplement shows the scoring intercepts and β coefficients that can be used to predict mortality in different patients with cirrhosis. An interactive CiMM scoring application (http://cimm.herokuapp.com/main) is available.

## Discussion

Better understanding of prognosis can frame patients’ preferences, help prioritize goals of care,^39^ and inform decision-making across many medical conditions. Machine learning models have greatly enhanced the accuracy of such predictions, but their black box analytics have limited their usefulness. The most useful models would combine the high predictive accuracy with the transparency and easy measurability of more traditional risk scores. Using cirrhosis as a test case, we found that a simple machine learning method enabled us to select electronic health record variables for a new blended CiMM. The CiMM had similar accuracy as less interpretable machine learning algorithms yet higher accuracy than the traditional MELD-Na severity score. The CiMM was more transparent than the machine learning models that helped select the variables and could be more easily applied in a variety of point-of-care clinical informatics infrastructures. Although we tested this approach in cirrhosis, it holds promise for improving prognostication across other medical conditions.

Multiple risk scores allow clinicians to estimate the risk of mortality in cirrhosis. The MELD-Na score is one of the most commonly used. Although originally developed to predict 90-day mortality, both primary care clinicians and specialists use it for general prognostication as well. The MELD-Na score had modest discriminative ability (AUC, 0.67) in our analysis. Our data show that relying on the MELD-Na score for prognostication beyond 90 days may not be ideal. Indeed, the 90-day window may be too short for most patients with cirrhosis, except for those on the liver transplant waiting list. For a risk prediction model to be clinically meaningful, it should predict events early enough to influence decisions and outcomes.^40^ The CiMM was able to accurately identify individuals at high risk of mortality at 1, 2, and 3 years (AUCs, 0.78, 0.76, and 0.71, respectively).

The variables selected by simple machine learning were consistent with those in the published literature, providing convergent validity to our results. However, some of these variables might not have been prioritized in alternative model-building strategies. One such example is the comorbidity (CirCom) score, which emerged as a strong predictor of mortality in this cohort of patients with advanced liver disease. These variables, including comorbidity, are easily available in electronic health records, rendering it feasible to implement the model at the patient and population levels. The CiMM can be incorporated into electronic health records from EPIC, Cerner Corporation, and others to easily automate and display prediction scores at the individual patient level. The CiMM can also be incorporated into population dashboards as part of quality improvement strategies. Within these population management systems, CiMM could identify high-risk patients for linkage to care, vigilant surveillance, and proactive care coordination. Matching the interventions with patients’ risk of mortality may allow more tailored approaches rather than 1-size-fits-all strategies,^41^ enhancing the overall effectiveness of quality improvement initiatives.

### Limitations

Our study has several limitations. We could not adjust for some factors, such as patients’ compliance with treatment, because these variables are difficult to operationalize from electronic health records. Some data were missing, but the rate of missingness was low, and data, when present, were imputed with the predicted values of a series of random-forests tree ensembles.^24^ Our study is limited to patients seen in the VA, most of whom were older men. Inclusion of a large racially and geographically diverse patient population may enhance generalizability, although future studies are needed to examine the external validity of CiMM in women and nonveterans before widespread deployment. The CiMM estimates prognosis in patients with cirrhosis and not the prognosis of cirrhosis per se. However, all-cause mortality is important in and of itself and represents an outcome that is most meaningful to patients with cirrhosis.^42^

## Conclusions

The promise of machine learning–based medical prognostication has been limited by implementation and interpretation challenges. We found that machine learning can help select important variables for more transparent risk scores while maintaining high rates of accuracy. The resultant blended CiMM performed better than the widely used MELD-Na score. If confirmed in other conditions, this blended approach could improve data-driven risk prognostication through the development of new scores that are more transparent and more actionable than machine learning and more predictive than traditional risk scores.

## Notes

Supplement. **eMethods 1.** Cohort Index Date **eMethods 2.** Imputation Approach **eMethods 3.** Machine Learning Models **eTable 1.** *ICD-9-CM* and *CPT* Codes and Drug Classes Used for Comorbidities Included in CirCom Score and Other Candidate Variables **eTable 2.** Baseline Characteristics of 107 939 Patients With Cirrhosis **eTable 3.** Sample Patients Profiles with 1- and 3-Year Mortality Based on Different Patient Characteristics **eTable 4.** Scoring Intercepts and β Coefficients for Predictors in Final Model Predicting Mortality in Patients With Cirrhosis **eFigure 1.** Discrimination Slopes for the Full Logistic With LASSO (Full Path LASS), Partial Path LASSO, and Gradient Boosting (XGB) Models **eFigure 2.** Calibration Slopes for the Full Logistic With LASSO (Full Path LASS), Partial Path LASSO, and Gradient Boosting (XGB) Models **eReferences.**
