# Supplementary material for: Development, Validation, and Evaluation of a Simple Machine Learning Model to Predict Cirrhosis Mortality
Source: JAMA Netw Open. 2020 Nov 3;3(11):e2023780. doi: 10.1001/jamanetworkopen.2020.23780 (PMC7610191; doi:10.1001/jamanetworkopen.2020.23780)
Supplement: Supplement. — eMethods 1. Cohort Index Date eMethods 2. Imputation Approach eMethods 3. Machine Learning Models eTable 1. ICD-9-CM and CPT Codes and Drug Classes Used for Comorbidities Included in CirCom Score and Other Candidate Variables eTable 2. Baseline Characteristics of 107 939 Patients With Cirrhosis eTable 3. Sample Patients Profiles with 1- and 3-Year Mortality Based on Different Patient Characteristics eTable 4. Scoring Intercepts and β Coefficients for Predictors in Final Model Predicting Mortality in Patients With Cirrhosis eFigure 1. Discrimination Slopes for the Full Logistic With LASSO (Full Path LASS), Partial Path LASSO, and Gradient Boosting (XGB) Models eFigure 2. Calibration Slopes for the Full Logistic With LASSO (Full Path LASS), Partial Path LASSO, and Gradient Boosting (XGB) Models eReferences. [file jamanetwopen-e2023780-s001.pdf]

Supplementary Online Content

Kanwal F, Taylor TJ, Kramer JR, et al. Development, validation, and evaluation of a simple machine learning model to predict cirrhosis mortality. *JAMA Netw Open*. 2020;3(11):e2023780. doi:10.1001/jamanetworkopen.2020.23780

eMethods 1. Cohort Index Date

eMethods 2. Imputation Approach

eMethods 3. Machine Learning Models

eTable 1. *ICD-9-CM* and *CPT* Codes and Drug Classes Used for Comorbidities Included in CirCom Score and Other Candidate Variables

eTable 2. Baseline Characteristics of 107 939 Patients With Cirrhosis

eTable 3. Sample Patients Profiles with 1- and 3-Year Mortality Based on Different Patient Characteristics

eTable 4. Scoring Intercepts and β Coefficients for Predictors in Final Model Predicting Mortality in Patients With Cirrhosis

eFigure 1. Discrimination Slopes for the Full Logistic With LASSO (Full Path LASS), Partial Path LASSO, and Gradient Boosting (XGB) Models

eFigure 2. Calibration Slopes for the Full Logistic With LASSO (Full Path LASS), Partial Path LASSO, and Gradient Boosting (XGB) Models

eReferences.

This supplementary material has been provided by the authors to give readers additional information about their work.

eMethods 1. Cohort Index Date

Because we wanted to develop a model that can be used in ambulatory settings, we defined our index date (for follow up) as the date of the first clinic visit (regardless of cirrhosis diagnosis at this visit) at or after patients met cohort entry criteria. Therefore, for a patient with 2 outpatient ICD codes for cirrhosis or complications (coded at the time of outpatient clinic visits), the index date was the first of these 2 visits. For a patient with one inpatient code (entered at the time of discharge from hospital) followed by one outpatient code (entered at the time of outpatient clinic visit); the outpatient visit was the index date. However, for a patient who had one inpatient visit with ICD code for liver disease with drug, his/her index date was the first outpatient visit for any reason that occurred after s/he met these criteria.

©2020 Kanwal F et al. *JAMA Network Open*.

eMethods 2. Imputation Approach

To impute the missing data, we used a non-parametric machine learning based imputation strategy—MissForest—that may have better accuracy relative to other imputation strategies such as Multiple Imputation (e.g., Multiple Imputation through Chained Equations).[1,2] MissForest predicts missing values through a series of non-parametric random forest tree ensembles. Briefly, the algorithm first makes an initial prediction of the missing value with a random forest fitting (first imputation iteration). Then, using the completely known data-matrix, a random-forest is trained with (y) representing the complete values observed from the predictor that contains missing values overall. Then, the missing values in that predictor (y) are predicted from the just-trained random forest model. This prediction is compared to the initial prediction of missingness. This process of training a new random forest on observed data plus the new predictions for the missing data and then making predictions of the truly missing data is repeated with a new prediction made until convergence. Convergence is assumed when the normalized root-mean-square-error (NRSME) of the prediction is minimized; when a new random forest prediction begins to increase the resulting NRMSE, the algorithm stops and the last random forest model predictions are used to impute the missing values.

One advantage of the MissForest approach is that a final dataset with a single prediction of the missing data values is the result. This makes MissForest more flexible for later machine learning methods like gradient boosting. In contrast, data-based multiple imputation methods require accounting for imputation of multiple datasets and then accounting for between-variance imputation. Accounting for between-imputation dataset variance in non-parametric machine learning models like gradient boosting remains unclear to the authors' knowledge.

©2020 Kanwal F et al. *JAMA Network Open*.

eMethods 3. Machine Learning Models

Gradient descent boosting creates a series of "boosted" decision trees of weaker predictors to create stronger final predictions. The final model was allowed to train up to 1,000 trees; however, optimization occurred at 127 trees. Additionally, a learning rate of 0.1 and a maximum depth of each tree of 7 (*i.e*., up to 7-way interactions) were identified as optimal during training.

The LASSO performs variable selection by first evaluating the magnitude of each predictor in a prediction model including the full set of predictors and then for each variable in the range full set of predictors, it adds a penalty (λ) to the prediction equation equal to the absolute value of smallest coefficient among all coefficients in the model at that stage of evaluation.[3,4] It then removes that variable and selects the remaining coefficients for the next iteration of penalty evaluation. This iterative loop continues until removal of predictors starts to increase prediction error. This process both selects the predictors that have the strongest influence on the outcome, while at the same time removes predictors that contribute little to the prediction. This facilitates a parsimonious prediction model that is more likely to be unbiased when predicting future data. Evaluating each predictor among all predictors available is customarily called the FULL pathway evaluation of predictors—as we refer to it here. We can also further constrain the prediction model to evaluate only a maximum number of most influential predictors (e.g., 10 or fewer predictors) and start the penalty process evaluation at the first iteration by selecting a penalty (λ) that is equal to the absolute value of the 11th most strongly predictive variable and starting the iteration through the remaining predictors after imposing that λ penalty. This results in a PARTIAL pathway as we do not evaluate all other predictors than the 10 most strongly influential predictors at the first iteration of the LASSO algorithm.

eTable 1. *ICD-9-CM* and *CPT* Codes and Drug Classes Used for Comorbidities Included in CirCom Score and Other Candidate Variables

**Cohort definition**	
	***ICD-9-CM* Codes**
Cirrhosis	571.2, 571.5, 571.6
Hepatocellular encephalopathy	070.71, 070.0, 070.2x, 070.4x, 070.6, 572.2, 348.3x
Ascites	789.5, 789.59
Esophageal varices	456.0, 456.1, 456.2x
HCC	155.0
Spontaneous bacterial peritonitis	567.23
Other decompensated cirrhosis	572.3, 572.4, 782.4, 572.8
**Candidate variables definitions**	
CirCom Score:	
(1) Nonmetastatic solid cancer	140.x-154.x, 156.x-165.x, 170.x-172.x, 173.79, 174.x-195.x, 199.x
(2) Metastatic cancer	196.x-198.x
(3) Hematologic cancer	200.x-208.x, 238.79, 273.3, 277.89
(4) Substance abuse other than alcoholism	292.x, 304.x, 305.x
(5) Epilepsy	345.x
(6) Acute myocardial infarction	410.x, 429.79
(7) Heart failure	428.x
(8) Peripheral arterial disease	440.x, 441.x, 443.81
(9) Chronic obstructive pulmonary disease	490, 491.0, 491.x, 492.x, 493.2x, 496
(10) Chronic kidney disease	585.x
Depression	296.2x, 296.3x, 293.83, 296.90, 296.99, 300.4, 309.0, 309.1, 311
Diabetes	249.x, 250.00, 250.02, 250.10, 250.12, 250.20, 250.22, 250.30, 250.32, 250.40, 250.42, 250.50, 250.52, 250.60, 250.62, 250.70, 250.72, 250.80, 250.82, 250.90, 250.92, 790.2x, 791.5, 791.6, V4585, V53.91, V65.46
Anxiety	300.0x, 300.10, 300.2x, 300.3, 300.89, 300.9
Severe infection:	
(1) Sepsis	995.91, 995.92, 785.52, 038.x

©2020 Kanwal F et al. *JAMA Network Open*.

(2) Infection	008.x, 009.x, 041.x, 480.x-487.x, 576.1
(3) Peritonitis	567.0
(4) Cellulitis	681.x, 682.x
Alcohol use	291.x, 303.x, 305.0x, 980.0, 357.5, 425.5, 535.3x, 790.3, V11.3
**Other therapies**	**CPT Codes**
Endoscopic variceal ligation	43244, 43205, 43400
Paracentesis	49080-49083
Transjugular intrahepatic portosystemic shunts	37182, 37183, C1040, C5283
**Medication classes**	**Drug class codes**
Antihypertensive	CV150, CV200, CV400, CV490, CV800, CV805, CV806
Analgesics	CN100-CN105
Antibiotic	AM000, AM110-AM120, AM150, AM200, AM250, AM300, AM350, AM400, AM550, AM600, AM650, AM700
Anti-depressive	CN600, CN601, CN602, CN609
Betablocker	CV100
Diuretic	CV700-CV704, CV709

ICD-9, CPT, and Drug Class Codes Used to Define the Advanced Liver Disease Cohort and Candidate Predictor Variables CirCom score uses a specific set of ICD-10 codes. We mapped the ICD-10 to ICD-9 codes to define conditions included in CirCom.

©2020 Kanwal F et al. *JAMA Network Open*.

eTable 2. Baseline Characteristics of 107 939 Patients With Cirrhosis

Characteristic	Data
**Age in years, mean (SD)**	62.7 (9.6)
**Race/ethnicity, N (%)**	
Black	19852 (18.4)
Hispanic	6376 (5.9)
White	71563 (66.3)
Other	3005 (2.8)
Missing	7143 (6.6)
**Sex, N (%)**	
Female	3623 (3.4)
Male	104316 (96.6)
**Marital status, N (%)**	
Divorced or separated	47981 (44.5)
Married	45792 (42.4)
Single or never married	14020 (13.0)
Missing	146 (0.1)
**Rural status, N (%)**	
Rural or highly rural	37140 (34.4)
Urban	69963 (64.8)
Missing	836 (0.8)
**Means test, N (%)**	
Copay exempt	30882 (28.6)
No longer required	40638 (37.7)
Pending or missing	14595 (13.5)
Required	21824 (20.2)
**Etiology of cirrhosis, N (%)**	
HCV infection alone	14286 (13.2)
HCV and alcohol	26011 (24.1)

©2020 Kanwal F et al. *JAMA Network Open*.

Alcohol alone	34112 (31.6)
Non-alcoholic steatohepatitis	29,140 (26.9)
HBV infection	3427 (3.2)
**HCV, N (%)**	
HCV RNA + w/out SVR at index	38708 (35.9)
HCV RNA+ w/ SVR at index	1589 (1.5)
No HCV	67642 (62.7)
**Aspartate aminotransferase/alanine aminotransferase (AST/ALT) ratio, N (%)**	
<2	83797 (77.6)
2 or higher	13699 (12.7)
missing	10443 (9.7)
**Laboratory test results, mean (SD)**	
Sodium level, mEq/L	137.7 (3.8)
Creatinine level, mg/dL	1.2 (1.0)
Bilirubin level, mg/dL	1.6 (2.7)
INR	1.4 (1.1)
Albumin level, g/dL	3.5 (0.7)
Platelet count, x 10^3/μL)	166.5 (92.7)
AST level	68.3 (134.4)
ALT level	60.2 (120.3)
Hemoglobin level, g/dL	13.0 (2.3)
**Cirrhosis complications, N (%)**	
Hepatic encephalopathy	21556 (20.0)
Ascites	21770 (20.2)
Varices (including variceal bleeding)	17631 (16.3)
Hepatocellular cancer	8150 (7.60)
Hepatorenal	412 (0.4)
Jaundice	13794 (12.8)
Spontaneous bacterial peritonitis	1106 (1.0)
**Mental health conditions, N (%)**	

©2020 Kanwal F et al. *JAMA Network Open*.

Depression	27464 (25.4)
Anxiety	9530 (8.8)
Alcohol use	39268 (36.4)
Drug use	16910 (15.7)
History of homelessness	9503 (8.8)
**Physical health conditions, N (%)**	
Diabetes	54137 (50.2)
Chronic obstructive pulmonary disease	17326 (16.1)
Myocardial infarction	1926 (1.8)
Peripheral arterial disease	4542 (4.2)
Epilepsy	4537 (4.2)
Heart Failure	11332 (10.5)
Cancer	18164 (16.8)
Chronic kidney disease	10872 (10.1)
Dialysis	2172 (2.0)
**CirCom Score*, N (%)**	
0	25649 (23.8)
1+0	28853 (26.7)
1+1	20362 (18.9)
3+0	5813 (5.4)
3+1	23807 (22.0)
5+0	109 (0.1)
5+1	3346 (3.1)
**Severe infection, N (%)**	13602 (12.6)
**Medication and treatment data, N (%)**	
Antihypertensives	65659 (60.8)
Analgesics	68835 (63.8)
Nonselective beta blockers	8931 (8.3)
Diuretics	48620 (45.0)
Anti-depressants	43293 (40.1)

©2020 Kanwal F et al. *JAMA Network Open*.

Antibiotics	48208 (44.7)
Endoscopic variceal ligation in the past year	1156 (1.1)
Paracentesis in the past year	2251 (2.1)
Transjugular intrahepatic portosystemic shunts in the past year	82 (0.1)
**Body mass index**	
<18.5	1915 (1.8)
18.5 to <25	26810 (24.8)
25 to <30	36546 (33.9)
30 or higher	40211 (37.3)
Missing	2457 (2.3)
**Smoking status**	
Current	39737 (36.8)
Former	26842 (24.9)
Nonsmoker	17792 (16.5)
Missing	23568 (21.8)
Pulse, mean (SD)	77.5 (15.5)
**Blood Pressure, mean (SD)**	
Systolic blood pressure,	129.9 (18.7)
Diastolic blood pressure	76.6 (10.6)
**Lipids, mg/dl, mean (SD)**	
High density lipoprotein	44.4 (15.6)
Low density lipoprotein	88.3 (34.1)
Total cholesterol	171.6 (33.6)
**Health care utilization**, **N (%)**	
Prior history of cirrhosis related hospitalization	
In the past year	27609 (25.6)
Any time before index	37041 (34.3)
Prior history of hospitalization from any cause	
In the past year	44143 (40.9
Any time before index	71570 (66.3)

©2020 Kanwal F et al. *JAMA Network Open*.

Hospitalization from primary diagnosis of cirrhosis or complications	10560 (9.8)
At least one emergency room visit in the past year	46450 (43.0)
# of outpatient visits in the past year	
0	4107 (3.8)
1	3062 (2.8)
2	2597 (2.4)
3+	98173 (91.0)
**Priority status,[40] N (%)**	
1-3	45150 (41.8)
4-5	46405 (43.0)
6-8	16038 (14.9)
Missing	346 (0.3)

*Circom: nonmetastatic cancer, metastatic cancer, hematologic cancer, substance abuse other than alcoholism, epilepsy, acute myocardial infarction, heart failure, peripheral arterial disease, chronic obstructive pulmonary disease, chronic kidney disease were pulled using most recent inpatient or outpatient diagnoses given in the 5 years before index date. Circom score was calculated by the algorithm[20] developed and validated by Jepsen et al. CirCom score uses a specific set of ICD-10 codes to define the conditions. We mapped these ICD-10 to ICD-9 codes to define conditions included in CirCom (as shown in Supplementary Table 1).

We used the Academy of Healthcare Research and Quality Clinical Classifications Software (CCS) to define the conditions that were not included in the CirCom score (such as diabetes, depression, anxiety, and alcohol use).

Abbreviations: CirCom, cirrhosis-specific comorbidity score; HBV, hepatitis B virus; HCV, hepatitis C virus

SI conversion factors: To convert albumin to g/L, multiply by 10.0; bilirubin to μmol/L, multiply by 17.104; creatinine to μmol/L, multiply by 88.4; hemoglobin to g/L, multiply by 10.0; platelet count to $\times 10^9$/L, multiply by 1.0; sodium to mmol/L, multiply by 1.0;

Unless otherwise indicated, data are expressed as number (percentage) of patients. Owing to missing data, percentages may not total 100.

©2020 Kanwal F et al. *JAMA Network Open*.

eTable 3. Sample Patients Profiles with 1- and 3-Year Mortality Based on Different Patient Characteristics

Age	Black race	Serum sodium (mEq/L)	Serum bilirubin (mg/dL)	Platelet count (10^3/μL)	Serum albumin (g/dL)	Hgb (g/dL)	AST/ ALT ratio >2	CirCom Score	HE	Ascites	HCC	Mortality risk	95% CI
**1-year mortality**													
55	1	135	2	150	3.5	12.2	0	1+0	0	1	0	0.12	0.12- 0.13
60	1	130	3	200	3.2	11.5	0	3+0	1	1	0	0.16	0.15- 0.17
60	0	135	3	150	3.3	12	0	1+1	0	0	0	0.18	0.17- 0.19
65	0	130	4	200	3	11	1	3+0	1	0	0	0.23	0.21- 0.25
70	0	125	5	200	2.5	10	1	3+1	0	0	0	0.45	0.43- 0.47
65	1	125	4	150	2.5	10.5	1	3+1	0	1	1	0.66	0.64- 0.68
**3- year mortality**													
55	1	135	2	150	3.5	12.2	0	1+0	0	1	0	0.34	0.20- 0.52
60	1	130	3	200	3.2	11.5	0	3+0	1	1	0	0.41	0.25- 0.58
60	0	135	3	150	3.3	12	0	1+1	0	0	0	0.44	0.28- 0.62
65	0	130	4	250	3	11	1	3+0	1	0	0	0.52	0.34- 0.69
70	0	125	5	200	2.5	10	1	3+1	0	0	0	0.75	0.58- 0.85
65	1	125	4	150	2.5	10.5	1	3+1	0	1	1	0.87	0.77- 0.93

Hgb – hemoglobin. HE – hepatic encephalopathy. HCC – hepatocellular cancer

©2020 Kanwal F et al. *JAMA Network Open*.

eTable 4. Scoring Intercepts and β Coefficients for Predictors in Final Model Predicting Mortality in Patients With Cirrhosis

**Model Structure**				
**Scoring Intercepts (Person Period Hazard)**	***a_j***			
Mortality in 1 Year	-.02			
Mortality in Year 2	.756			
Mortality in Year 3	.673			
Mortality in Year 4	.628			
Mortality in Year 5	.638			
Mortality in Year 6	.652			
Mortality in Year 7	.642			
Mortality in Year 8	.679			
**Risk Predictors**	**B**	**Relative Risk**	**95% CI lower limit**	**95% CI upper limit**
Age	.039	1.04	1.03	1.04
Black race	-.158	.87	.85	.89
Serum sodium (meq/l)	-.018	.98	.98	.99
Serum bilirubin (mg/dl)	.051	1.05	1.04	1.05
Platelet count (per 50/ml)	-.062	.95	.94	.95
Serum albumin (mg/dl)	-.513	.63	.62	.64
Hemoglobin (g/dl)	-.071	.94	.94	.94
AST to ALT ratio ≥ 2	.269	1.26	1.24	1.29
Hepatic encephalopathy	.208	1.2	1.18	1.22
Ascites	.304	1.3	1.28	1.33
Hepatocellular cancer	.949	2.18	2.13	2.23
CirCom score 1+0 (ref = CirCom = 0)	.276	1.27	1.24	1.3
CirCom score 1+0	.487	1.52	1.48	1.56
CirCom score 3+0	.169	1.16	1.12	1.21
CirCom score 3+1	.649	1.73	1.69	1.77
CirCom score 5+0	.994	2.26	1.85	2.71
CirCom score 5+1	1.321	2.84	2.74	2.94

©2020 Kanwal F et al. *JAMA Network Open*.

	Number of visits ≥ 3	-.291	.77	.75	.79

We removed the intercept term when fitting discrete time models, and each person-period has a coefficient associated with the hazard of death in that time period. Using the first year as example, the expected person-period hazard for a patient would be aj=1=-.02 and the result from this estimate is an adjustment in the predicted risk by 1/(1 + exp(-aj=1)) = 1/(1 + exp(-(-.02)) = .49. This probability of mortality in 1 year from cohort entry may seem quite high (~50/50 chance); yet, we note that many of the predictors are "protective" in the sense that they reduce risk to a value lower than .49. It is also important to note that that these person-period hazards are conditional probabilities in and of themselves as the probability of death in 2 years is conditional on the probability that one survives through 1 year, and so forth.

©2020 Kanwal F et al. *JAMA Network Open*.

eFigure 1. Discrimination Slopes for the Full Logistic With LASSO (Full Path LASS), Partial Path LASSO, and Gradient Boosting (XGB) Models

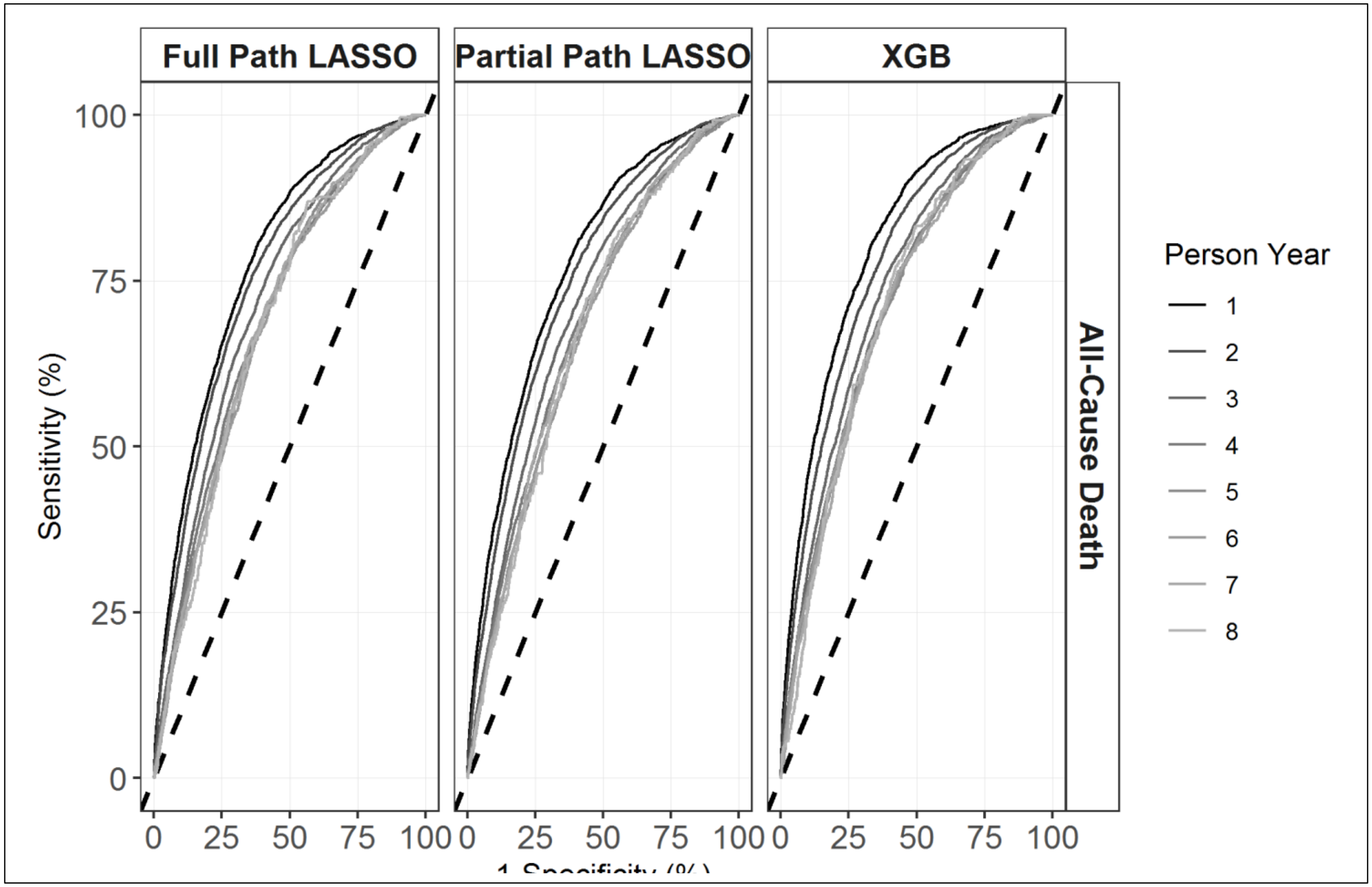

©2020 Kanwal F et al. *JAMA Network Open*.

eFigure 2. Calibration Slopes for the Full Logistic With LASSO (Full Path LASS), Partial Path LASSO, and Gradient Boosting (XGB) Models

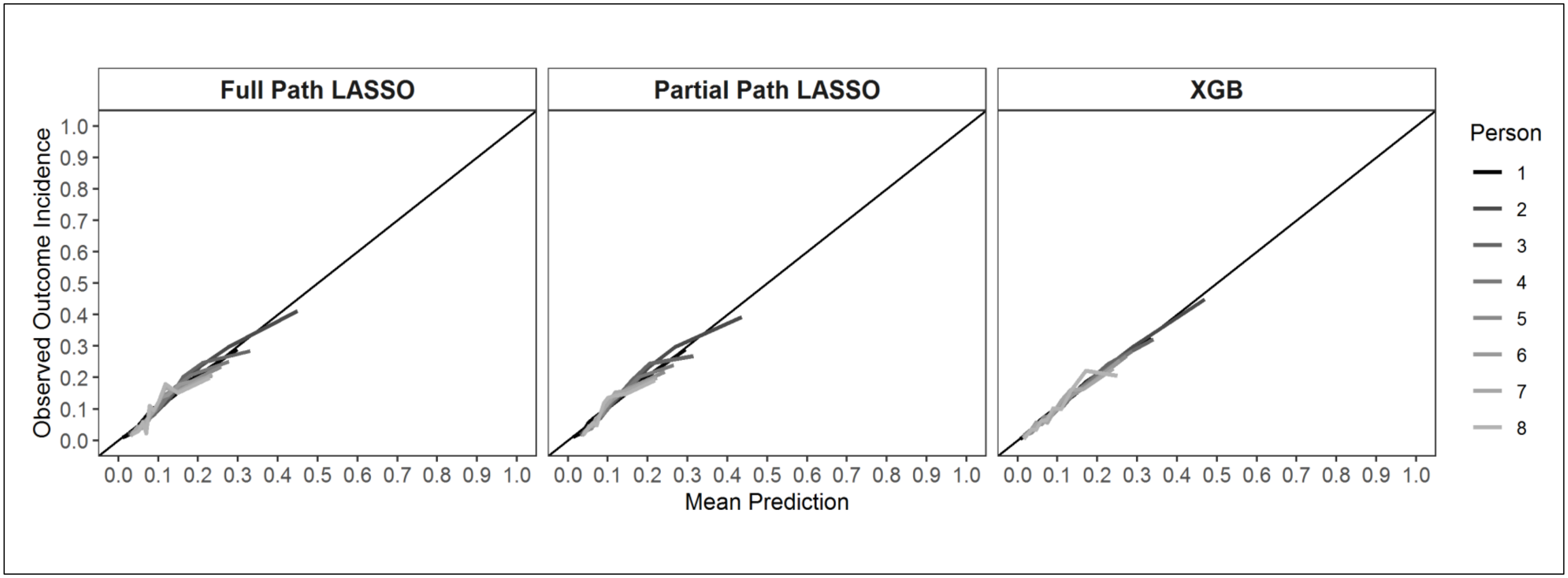

©2020 Kanwal F et al. *JAMA Network Open*.

eReferences.

1. Daniel J. Stekhoven, Peter Bühlmann, MissForest—non-parametric missing value imputation for mixed-type data, *Bioinformatics*, Volume 28, Issue 1, 1 January 2012, Pages 112–118
2. Anoop D. Shah, Jonathan W. Bartlett, James Carpenter, Owen Nicholas, Harry Hemingway, Comparison of Random Forest and Parametric Imputation Models for Imputing Missing Data Using MICE: A CALIBER Study, *American Journal of Epidemiology*, Volume 179, Issue 6, 15 March 2014, Pages 764–774
3. Hastie T, Tibshirani, R. Friedman, J. The Elements of Statistical Learning: Data Mining, Inference, and Prediction. In: Springer-Verlag; 2009: https://web.stanford.edu/~hastie/ElemStatLearn/
4. Muthukrishnan R, Rohini R. LASSO: A feature selection technique in predictive modeling for machine learning. Paper presented at: 2016 IEEE International Conference on Advances in Computer Applications (ICACA); 24-24 Oct. 2016, 2016. https://ieeexplore.ieee.org/abstract/document/7887916

©2020 Kanwal F et al. *JAMA Network Open*.
